# Elevated temperature and browning increase dietary methylmercury, but decrease essential fatty acids at the base of lake food webs

**DOI:** 10.1038/s41598-021-95742-9

**Published:** 2021-08-19

**Authors:** Pianpian Wu, Martin J. Kainz, Fernando Valdés, Siwen Zheng, Katharina Winter, Rui Wang, Brian Branfireun, Celia Y. Chen, Kevin Bishop

**Affiliations:** 1 Department of Aquatic Sciences and Assessment, Swedish University of Agricultural Sciences, Uppsala, Sweden; 2 Department of Biological Sciences, Dartmouth College, Hanover, USA; 3 WasserCluster Lunz- Biologische Station, Lunz Am See, Austria; 4 Department of Biomedical Research, Danube University Krems, Krems, Austria; 5 Uppsala University, Uppsala, Sweden; 6 College of Environmental Science and Engineering, Tongji University, Shanghai, China; 7 Department of Biology, Western University, London, Canada

**Keywords:** Biogeochemistry, Environmental sciences

## Abstract

Climate change scenarios predict increases in temperature and organic matter supply from land to water, which affect trophic transfer of nutrients and contaminants in aquatic food webs. How essential nutrients, such as polyunsaturated fatty acids (PUFA), and potentially toxic contaminants, such as methylmercury (MeHg), at the base of aquatic food webs will be affected under climate change scenarios, remains unclear. The objective of this outdoor mesocosm study was to examine how increased water temperature and terrestrially-derived dissolved organic matter supply (tDOM; i.e., lake browning), and the interaction of both, will influence MeHg and PUFA in organisms at the base of food webs (i.e. seston; the most edible plankton size for zooplankton) in subalpine lake ecosystems. The interaction of higher temperature and tDOM increased the burden of MeHg in seston (< 40 μm) and larger sized plankton (microplankton; 40–200 μm), while the MeHg content per unit biomass remained stable. However, PUFA decreased in seston, but increased in microplankton, consisting mainly of filamentous algae, which are less readily bioavailable to zooplankton. We revealed elevated dietary exposure to MeHg, yet decreased supply of dietary PUFA to aquatic consumers with increasing temperature and tDOM supply. This experimental study provides evidence that the overall food quality at the base of aquatic food webs deteriorates during ongoing climate change scenarios by increasing the supply of toxic MeHg and lowering the dietary access to essential nutrients of consumers at higher trophic levels.

## Introduction

With climate change, warming is expected^1^ along with altered precipitation patterns in freshwater ecosystems^2^. Increase in precipitation often leads to increasing dissolved organic carbon (DOC) concentrations and water colour in boreal aquatic ecosystems^3^. Significant increases in the amount of terrestrially-derived dissolved organic matter (tDOM) has already occurred with a doubling of DOC during the past 25 years in some systems, often termed browning (or brownification)^4,5^. Warming and browning of aquatic ecosystems have important consequences for both ecosystem function and the dietary quality of aquatic resources, starting at the base of the food web. Despite there being a widely accepted recognition that increases in temperature and tDOM will be occurring, there is little knowledge on how effects of warming and/or browning will affect the dietary quality of aquatic resources, particularly from an ecotoxicological point of view. Climate change may present both changed risk levels to organisms from environmental pollutants, such as the potent neurotoxin, mercury (Hg) and its highly bioavailable form methylmercury (MeHg)^6^, as well as altered benefits from essential dietary compounds, such as polyunsaturated fatty acids (PUFA), which are crucial for somatic growth, development, and survival of biota^7,8^. Both the bioconcentration of MeHg and biosynthesis of PUFA at the base of the food web greatly impact the health and survival of organisms at higher trophic levels^9,10^.

So far, there have been claims of changes in MeHg bioaccumulation due to climate warming or browning. For example, experimental and empirical work have shown warming leading to increases of fish Hg^11,12^, as well as similar temperature effects on MeHg bioaccumulation in macroinvertebrates in experimental estuary ecosystems^13,14^. No evidence, however, has been presented about temperature effects on MeHg at the base of the food web in freshwaters, even though this is an essential link in Hg bioaccumulation^9^. The influence of increasing tDOM has been tested on zooplankton in brackish waters which resulted in increased MeHg bioaccumulation^15^. On the other hand, a study conducted in Arctic lakes showed that higher DOC concentrations in lakes inhibited bioaccumulation of both total Hg and MeHg in aquatic microbiota^16^, and there was an indication that DOC reduces fish Hg bioaccumulation^17^. A similar finding that high DOC levels lower MeHg bioavailability to stream macroinvertebrates and fish was observed in stream ecosystems from the Northeast United States^18^. As for the climate effects on the dietary quality at the base of the food web, only a few studies have been conducted. A significant loss of phytoplankton diversity and evenness was noted under warming and temperature fluctuations, leading to dominance by Cyanobacteria^19^. This suggests a loss of dietary quality with decreasing omega-3 PUFA resulting in nutritional deterioration for higher consumers.

The effects of browning and the simultaneous influence of increasing temperatures on MeHg and PUFA at the base of the food web have not been examined yet. Therefore, we conducted an outdoor mesocosm experiment to investigate how the MeHg content and dietary quality (as assessed by PUFA) would change in organisms at the base of the aquatic food web with increased water temperature and/or increased supply of terrestrial organic matter. We predicted that the combined effects of warming and browning would increase the MeHg content and decrease the dietary quality^19^. We also expected that zooplankton biomass would increase due to higher algal biomass that is caused by higher temperature and browning, with the latter also increasing the nutrient supply^20,21^.

We used 24 polyethylene containers (400 L each) as experimental enclosures (mesocosms) for the experiment to test the effect of four treatments. Six containers were used to mimic ambient temperature and light conditions (controls; C treatment), whereas in another treatment we increased the temperature by 3 °C above ambient^22^ (temperature increase; T treatment). In the browning (B) treatment we added an extracted tDOM solution on a weekly basis to create a two-fold increase in DOC concentrations, which was similar to the lower range of DOC concentrations found in Scandinavian freshwater ecosystems^23^. This mimics a possible future scenario of episodic/seasonal floods with increased inputs of allochthonous organic compounds to lakes^24^. The final treatment combined the T and B treatments (mixed conditions; TB treatment) to represent another likely future scenario of simultaneous warming and browning within a century at similar nutrient levels.

The temperature was steered by a computerized system that maintained the temperature in the heated treatment 3 °C above the controls. Each treatment was replicated six times. The nutrient levels and spiked MeHg concentrations were identical among treatments throughout the study. Phytoplankton and zooplankton were added in equal mass volume to each enclosure at the beginning of the study. Water samples were taken weekly for aqueous MeHg, DOC, and nutrient analysis from 2 July to 13 August 2018. Organisms of two different size classes were sampled: (a) seston (0.7–40 μm), which is considered the most consumed particle size range by zooplankton^25,26^; and (b) microplankton (40–200 μm), which contained a mix of algae and very few small-sized zooplankton. Thus, it was possible to capture the temperature and browning effects at the base of the food web, specifically the algal-animal interface^27^. Seston and microplankton were sampled at the end of the experiment (14 August to 16 August 2018) for analysis of biomass, as well as MeHg and FA contents. We normalized for ambient (control) conditions, calculated a Cohen’s d effect size for each treatment, and used a completely randomized and fully replicated factorial analysis of variance (ANOVA) to assess the expected direction and level of change in seston and microplankton biomass, MeHg content (both measured concentration and calculated biomass burden) and PUFA.

## Results and discussion

**Figure 1:**
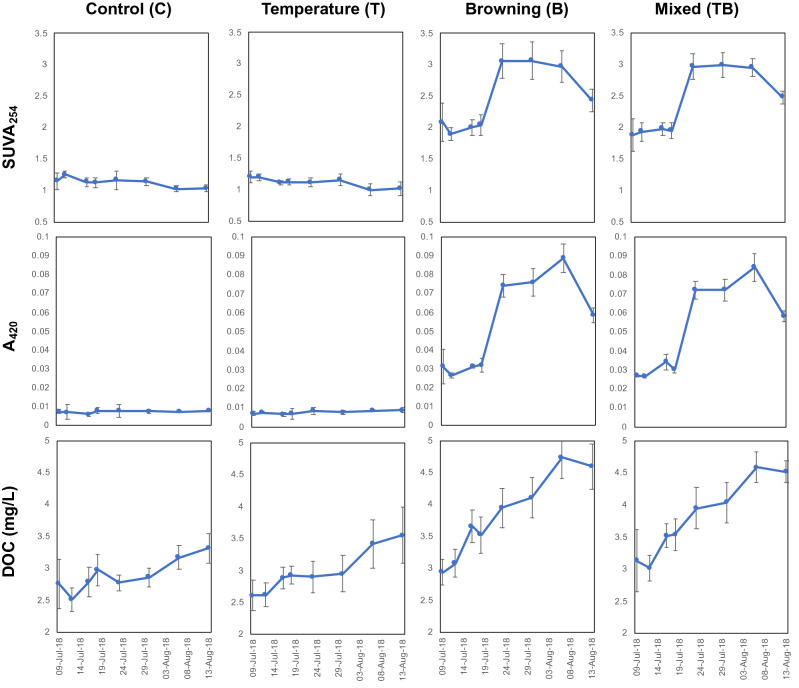
Temporal dynamics in water column aromaticity. Top to bottom: Specific UV absorbances at 254 nm (SUVA_254_), water colour indicated by absorbance at 420 nm (A_420_), and dissolved organic carbon (DOC) concentrations filtered through 0.2 µm. All marked with means ± 1 s.d.

The additions of extracted tDOM solution gradually darkened mesocosm water in the browning (B) and mixed treatments (TB) as determined by colour (absorbance at 420 nm, A_420_). This mainly resulted from an increase in more aromatic DOM compounds (indicated by specific UV absorbance at 254 nm, SUVA_254_) (Fig. 1). The increase in DOC concentrations (filtered through 0.2 μm) was less than the increase in water colour (Fig. 1), indicating that tDOM additions increased the proportion of organic matter molecules in the mesocosm water with high aromaticity^28^.

Methylmercury in the ambient lake water was below detection limit. The initial MeHg spike raised the aqueous (< 0.2 μm) MeHg concentration. Additional MeHg spikes were made each week, but the mesocosm MeHg still decreased slowly across all treatments during the experiment (Fig. S4 and S5). This could result from uptake of dissolved MeHg by seston, and/ or photodegradation of aqueous MeHg (Gilmour et al. 1998). Towards the end of the experiment, the aqueous MeHg concentrations were highest in the mixed treatment mesocosms compared to other treatments (*p* < 0.05) (Table 1).

**Table 1:** Summary of analysed methylmercury and fatty acids from  sampling at the end of the mesocosm experiment. Contents (means ± 1 s.d.) of methylmercury (MeHg), lipids and polyunsaturated fatty acids (PUFA) in aqueous (< 0.2 μm), seston (0.7–40 μm), microplankton (40–200 μm), and zooplankton (> 55 μm) as well as plankton biomass at the end of the mesocosm experiment. “n” = number of replicates of each treatment. Fatty acids are determined as fatty acid methyl esters (FAME).

Measurements	Treatments			
	Control (n = 6)	Temperature (n = 6)	Browning (n = 6)	Mixed (n = 6)
Aqueous MeHg (pg/L)	4.61 ± 3.50	4.36 ± 2.35	7.75 ± 3.46	11.36 ± 4.96
MeHg, unfiltered (pg/L)	41.72 ± 8.53	35.02 ± 5.02	42.14 ± 6.20	40.11 ± 13.52
MeHg in seston (ng/g, d.w.)	3.05 ± 1.77	4.01 ± 2.44	2.19 ± 0.55	3.05 ± 0.76
Biomass in seston (mg/L)	2.91 ± 0.50	8.42 ± 5.21	8.89 ± 2.44	6.83 ± 1.22
MeHg burden in seston by volume (pg/L)	8.81 ± 5.46	24.64 ± 7.35	19.48 ± 7.23	20.55 ± 4.99
Total lipids in seston (mg/g, d.w.)	93.41 ± 33.31	81.35 ± 35.69	52.25 ± 15.46	55.9 ± 23.09
n-3 FA in seston (μg FAME/g, d.w.)	4.18 ± 1.41	3.77 ± 1.78	2.07 ± 0.73	2.1 ± 0.65
n-6 FA in seston (μg FAME/g, d.w.)	2.81 ± 1.1	3.36 ± 1.9	1.25 ± 0.4	1.33 ± 0.6
MeHg in microplankton (ng/g, d.w.)	3.36 ± 1.26	2.89 ± 0.89	4.56 ± 1.65	4.45 ± 2.37
Biomass in microplankton (mg/L)	7.29 ± 3.18	9.51 ± 3.83	9.68 ± 2.75	9.93 ± 3.25
MeHg burden in microplankton by volume (pg/L)	22.04 ± 7.64	25.47 ± 6.23	41.41 ± 8.92	38.0 ± 9.4
Total lipids in microplankton (mg/g, d.w.)	34.54 ± 4.01	43.76 ± 16.13	51.94 ± 11.21	36.13 ± 4.9
n-3 FA in microplankton (μg FAME/g, d.w.)	3.51 ± 0.86	5 ± 2.17	8.74 ± 2.56	5 ± 1.18
n-6 FA in microplankton (μg FAME/g, d.w.)	2.29 ± 1.01	4.1 ± 2.1	4.67 ± 0.67	3.51 ± 0.99
MeHg in zooplankton (ng/g, d.w.)	8.52 ± 3.05	7.09 ± 1.28	8.69 ± 2.55	8.38 ± 2.41
Biomass in zooplankton (mg/L)	1.37 ± 0.34	1.76 ± 0.34	4.95 ± 1.51	3.75 ± 1.18
MeHg burden in zooplankton by volume (pg/L)	9.66 ± 2.77	11.35 ± 2.04	36.61 ± 8.4	28.84 ± 8.08

**Figure 2:**
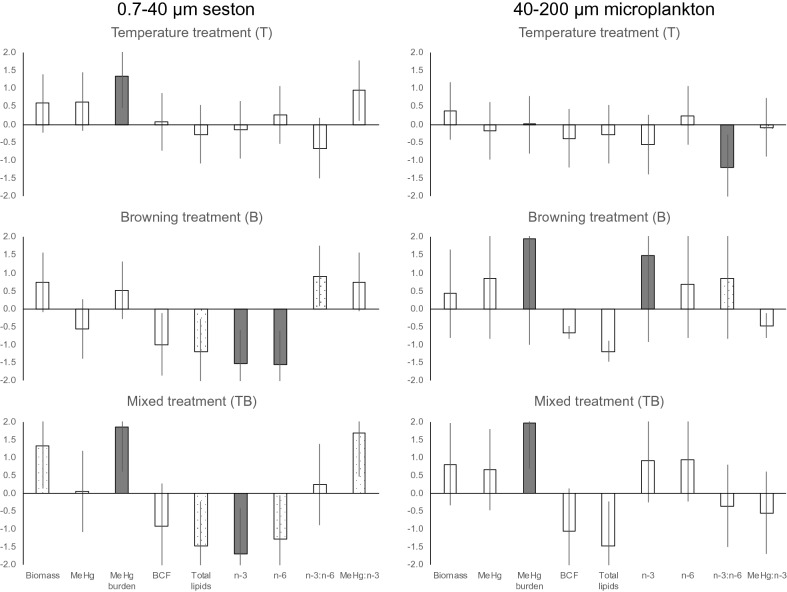
Cohen’s d effect sizes for response variables based on results from sampling at the end of the mesocosm experiment. Response variables: seston and microplankton biomass, MeHg content and bioconcentration factors, as well as fatty acids. Mean effect sizes (± higher/lower confidence intervals; ± CI) of 0.7–40 μm seston were analysed at the end of the experiment for biomass, MeHg concentrations, MeHg burden (MeHg in seston biomass per unit volume) and BCF (log transformed ratio of MeHg in seston versus aqueous MeHg; < 0.2 μm), total lipids, omega-3 (n-3) and omega-6 (n-6) PUFA concentrations, as well as ratios of MeHg:n-3 PUFA, and n-3:n-6 in seston (left panel). Effect sizes of the same response variables for 40–200 μm microplankton sampled and analysed at the end of the experiment are shown in the right panel. Treatments included in the mesocosm experiment: increased temperature (3 °C; T; upper panel), browning (B; continuous increase of tDOM solution; middle panel), and the interaction of temperature and browning (mixed treatment; TB; lower panel). The bars show how a future scenario will push ( ±) each response compared with the ambient situation Control (C). The y axis states Cohen’s d effect sizes (d). Dark grey bars indicate statistically significant (*p* < 0.05) and large effect sizes (d ≥ 0.8) that are more meaningful^28^ compared to unfilled bars with smaller effect sizes (d < 0.8). Dotted bars indicate responses with large effect sizes (d ≥ 0.8) but with less statistical significance (*p* < 0.10).

Our future scenario for organisms at the base of food webs in aquatic ecosystems, with temperature elevated by 3 °C and an increase of the tDOM, revealed major effects on MeHg burden, total lipids, and PUFA contents for both size fractions at the base of the food web. This suggests that both potential food risk and dietary quality were strongly influenced by increased temperature and browning (Fig. 2). The influence of increased temperature on seston MeHg burden was more pronounced compared to microplankton, contrasting with the influence of browning that was significant on seston and microplankton PUFA contents, but not seston MeHg burden. This indicated that the uptake of MeHg differs from the retention of essential dietary compounds when plankton is exposed to warming or browning scenarios. Under combined warming and browning treatments, increased biomass with constant or increasing aqueous MeHg concentrations resulted in an increased MeHg burden for organisms of both size fractions, and thus an increase in the amount of MeHg available for uptake at the base of food web.

Combined increases in temperature and browning increased seston biomass significantly in the mixed treatment (TB) (F_1,20_ = 9.84, *p* < 0.01, Table 2; Fig. 2), and a simultaneous increase of MeHg burden was observed, while aqueous MeHg concentrations remained stable (Fig. 2). Higher seston biomass and MeHg burden in seston in the temperature (T) and browning (B) treatments indicate that organisms in both treatments increased aqueous MeHg transfer to the base of the food web relative to the Control (C), even though aqueous MeHg concentrations were similar. The exact mechanisms remain speculative: it may be due to aqueous MeHg being increasingly adsorbed to increasing seston biomass under temperature increase, which also provides more binding sites for MeHg. Another possibility is shading by tDOM in the water column allowing aqueous MeHg to avoid photodegradation longer^29^, which likely preserved MeHg concentrations in the browning treatments.

**Table 2:** Statistics from the factorial ANOVA of the response variables based on results from sampling at the end of the mesocosm experiment. Response variables: seston and microplankton biomass, MeHg and MeHg burden, and bioconcentration factors, total lipids, and omega-3 polyunsaturated fatty acids (n-3 PUFA), omega-6 polyunsaturated fatty acids (n-6 PUFA), under the 2-factor Treatment (T, B, and TB). The values of *p* were adjusted for multiple comparisons (FDR *p* values), and the *p* values in bold indicate statistical significance level *p* < 0.05.

	*df*	Biomass		MeHg						Lipids and PUFA								MeHg:n-3	
		*F*	*p*	MeHg		MeHg burden		BCF		Total lipids		n-3		n-6		n-3:n-6		*F*	*P*
				*F*	*p*	*F*	*p*	*F*	*P*	*F*	*p*	*F*	*p*	*F*	*p*	*F*	*p*		
***0.7–40 μm seston***																			
***Treatment***	3,20																		
T		2.05	0.27	2.51	0.45	10.65	** < 0.05**	0.03	0.94	0.47	0.57	0.15	0.81	0.44	0.59	2.66	0.19	5.33	0.13
B		3.32	0.17	0.55	0.45	1.62	0.29	5.83	0.20	8.44	< 0.10	14.05	**0.01**	14.42	** < 0.01**	5.04	< 0.10	3.40	0.16
TB		9.84	< 0.10	0.00	0.92	8.11	** < 0.05**	0.08	0.24	0.13	< 0.10	0.19	** < 0.05**	0.24	0.06	1.60	0.80	0.20	< 0.10
***40–200 μm microplankton***																			
***Treatment***	3,20																		
T		0.84	0.55	0.19	0.82	< 0.01	< 1	0.90	0.50	0.61	0.51	1.90	0.21	0.38	0.55	8.51	** < 0.05**	0.04	0.84
B		1.10	0.53	4.23	0.16	23.01	** < 0.01**	2.61	0.49	1.35	0.35	13.20	** < 0.01**	2.79	0.23	4.22	< 0.10	1.29	0.54
TB		0.54	0.63	0.07	0.89	1.06	** < 0.05**	0.11	0.49	8.82	0.79	11.39	0.20	7.68	0.23	< 0.00	0.55	5.50	0.54

The MeHg burden in microplankton increased under the browning (B and TB) treatment (*p* < 0.01, Table 1). However, the treatment condition and their interaction had no significant additive effect on microplankton biomass, BCF, or aqueous MeHg concentrations. This suggests a consistent aqueous MeHg transfer across the algal-animal interface, which is critical for further bioaccumulation and trophic transfer^9^.

It is important to note that seston MeHg bioconcentration factors (BCF) showed no significant change in any of the treatments, even though in the browning (B) and mixed treatments (TB) the BCF was lower than that of the Control (C). Previous studies have shown that increases in DOC inhibit biological uptake of MeHg^6,16^, likely due to more binding sites for MeHg that reduce uptake by biota directly from the aqueous phase^30^. Our experimental results with elevated MeHg burden in plankton when DOC increased in the mixed treatment (TB) contradicted with this previous work that suggests decreasing MeHg bioavailability to the food web when DOC increases. We would suggest instead that increasing DOC content does not offset the general pattern of higher MeHg uptake by plankton under higher temperature. Our results revealed that temperature and browning factors have implications for MeHg burden in seston at the base of the food web, as well as MeHg burden in microplankton. This will have direct bearing on consumers at higher trophic levels.

However, even as MeHg burdens increased, the concentration of total lipids, omega-3 (n-3) PUFA, and omega-6 (n-6) PUFA in seston decreased in browning (B) and mixed treatments (TB) (Table 2). The PUFA decrease under the browning treatment (B) was most significant for n-3 PUFA (F_1,20_ = 14.05, *p* = 0.01; Fig. 2, Table 2) and n-6 PUFA (F_1,20_ = 14.42, *p* < 0.01; Fig. 2, Table 2). These changes indicate low diet quality that may subsequently lower somatic growth and reproduction of zooplankton and other consumers, likely due to altered taxonomic seston composition^19,31^. Nevertheless, we were able to observe microplankton PUFA increases in the browning treatment (B) regarding n-3 PUFA (F_1,20_ = 13.20, *p* < 0.01; Table 2, Fig. 2). Such increase of PUFA in microplankton may not all become readily available for subsequent nutrient transfer to the next trophic level, considering that the microplankton size fraction (40–200 μm) is largely inaccessible for zooplankton^25,32^.

To get a better understanding of the change in fatty acid quality and relevant dietary benefit/risk quantitatively, we explored the n-3:n-6 PUFA ratios, which is useful in assessing the relative contribution of autochthonous versus allochthonous matter in food sources^33^, as well as the MeHg:n-3 PUFA ratio in both seston and microplankton (Fig. 2, Table 2). The ratio of MeHg:n-3 PUFA in seston increased slightly in the mixed treatment (TB) (F_1,20_ = 0.2, *p* < 0.10; Table 2, Fig. 2), suggesting relatively elevated dietary exposure to MeHg, yet decreased supply of PUFA to aquatic consumers from seston. The n-3:n-6 were the lowest in the temperature treatment (T) for both seston and microplankton, but they increased in the browning treatment (B) (Fig. 2), indicating enhanced dietary availability of n-3 PUFA for aquatic consumers under tDOM addition. Similarly, the isopod *Asellus* utilized algal resources best when feeding on algae under terrestrial organic matter dominated conditions^34^. However, this result seems to contrast with findings from other previous studies where terrestrial taxa contain higher n-6 PUFA that differentiate them from aquatic OM that contains relatively more n-3 PUFA^35^. While additional nutrients associated with tDOM input in the browning treatment could have stimulated autochthonous OM production in the experiment, the increased SUVA suggests the opposite. Alternatively, the higher n-6 PUFA from sites with more terrestrial inputs in other studies could be related to particulate organic carbon which was not added in our study^36^.

**Figure 3:**
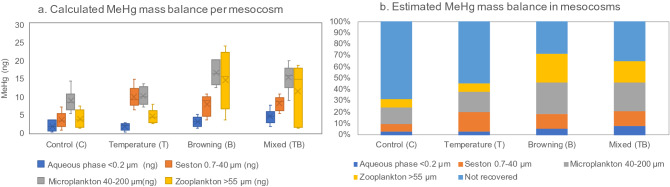
Estimated MeHg mass balance per mesocosm (**a**) and MeHg distribution across aqueous and biological phases (**b**) across treatments based on average estimations. Aqueous phase (dark blue), seston (orange), microplankton (grey), and zooplankton (yellow). “Not recovered” in light blue colours represent the proportion of MeHg that was not retrieved in either aqueous phase or solid phase at the end of the mesocosm experiment.

The MeHg mass balance was estimated from measurements on aqueous MeHg, seston, microplankton, and zooplankton (Fig. 3a). This led to an estimated distribution of the MeHg mass in the mesocosms relative to the total amount of spiked MeHg (Fig. 3b). A larger proportion was recovered in the brownified (B and TB) mesocosms (65% to 70% recovery of the spiked MeHg, relative to the control and temperature treatments (C and T) where 40% and 30% of the respective MeHg spike was recovered in the water column or biota (Fig. 3b). This suggests a loss of MeHg that was larger in the non-brownified (C and T) treatments. This could be due to the darker water colour attenuating light penetration in the open mesocosms, decreasing dissolved MeHg photodegradation processes by UV light^37^. Within browning and mixed treatments, there is also a possibility of higher microbial Hg methylation, stimulated by organic matter remineralization^38^. Another possibility of not-recovered MeHg could be due to aqueous MeHg lost to the walls of mesocosms^39^, but it is not clear why such losses would be lower in the brownified treatments. The proportions of MeHg distributed to seston under all treatment conditions, as well as microplankton in the mixed treatment (Fig. 3b), are substantially higher compared to the control (C) condition, presumably driven by plankton biomass increase. The microplankton comprised mostly of filamentous algae is, however, not readily bioavailable for consumers at higher trophic levels due to particle size limitations^25,32^.

Mesocosms have higher surface to volume ratios than natural systems and the “wall-effect” is common to mesocosm experiments^40^. The “wall effect” in this current study may have increased the amount of microplankton relative to what might be expected in nature. We observed that this effect was comparable across the different treatments so we do not expect the effects of the treatments compared to controls (Fig. 2) to change from the current conclusions. However, the “wall effect” could change the mass balance such that there would be a lower mass percent of the total MeHg mass in the microplankton. In addition, the “wall effect” could have also resulted in biomass dilution of the MeHg concentrations in the microplankton fraction shown in Table 1, resulting in lower MeHg concentrations comparing to where no or less “wall effect” occurred.

Here we demonstrate that the expected biomass increases created by increased temperature and browning in the combined (TB) treatment was accompanied by contrasting changes in MeHg and PUFA content, with increasing MeHg burden and decreasing contents of highly required dietary PUFA in both the seston and microplankton. The sensitivity of the base of the food web to large-scale climate changes has important consequences for all consumers at higher trophic levels. This was apparent from the responses (or lack thereof) for seston biomass, MeHg burden and lipid contents, as well as PUFA to the temperature and tDOM increases in the separate T and B treatments (Fig. 2, Table 2): In the temperature (T) treatment, it was only the MeHg burden in the seston that increased significantly, while in the browning (B) treatment it was only the PUFA that decreased significantly in the seston.

The microplankton fraction maintained its biomass, MeHg and PUFA contents under the increased temperature scenario, suggesting that larger size phytoplankton is less responsive to temperature hikes than seston. However, microplankton MeHg responded to tDOM addition. The browning scenario increased the lipid and PUFA contents in microplankton (Table 2), corresponding to higher content of n-3 PUFA under tDOM-rich conditions^34^. Elevated MeHg burden in the mixed (TB) treatment was a common response for seston and microplankton in the mixed treatment (Fig. 2), despite their different responses with respect to MeHg and PUFA by adding tDOM alone (B treatment). Consequently, we conclude that climate change scenarios with increasing lake temperature and browning affect MeHg uptake and dietary PUFA at the aquatic plant/animal interface: A warming scenario without browning increases aqueous MeHg uptake by seston, thus increasing dietary MeHg for consumers, while lake browning increases PUFA contents in microplankton, which may not be readily available for zooplankton.

Our study was designed to compare effects of two climate change variables to ambient (control) conditions, in order to simulate responses at the base of aquatic food webs in subalpine lake ecosystems. We investigated these effects under subalpine conditions that are highly prone to climate change effects, and relatively tDOM-poor compared to many other limnic systems. More and more lakes and reservoirs, especially those in boreal regions, are facing both temperature increase (with fewer winter ice days) and browning (higher tDOM content in water)^5^. Little has been done to observe the combination of the two climate change factors simultaneously, although warnings have been given about the threats from warming^11^ as well as the role of tDOM for MeHg bioavailability in some situations^41,42^. Hence, we are here able to add a mechanistic understanding of the combined effects of warming and browning to the observations on MeHg bioavailability and lipid content under climate change. Moreover, we connect predicted climate-change factors with environmental consequences in aquatic ecosystems. This places the basal food web investigations of the present study in the scope of global change processes, thereby providing a scenario for better understanding the biogeochemical cycles of MeHg and essential nutrient lipids in future aquatic ecosystems.

## Materials and methods

### Experimental setup and treatments

The mesocosm facility at WasserCluster Lunz (47°51′N, 15°01′E, elevation: 608 m) was utilized for this experiment. Modelled ecosystems with oligotrophic subalpine lake water from Lake Lunz^41^ were constructed in thermally insulated high-density polyethylene cylinder mesocosms (n = 24, 400 L volume, 1 m × 0.74 m Ø). The subalpine lake water is characterized by high pH (> 8), low DOC (< 2 mg/L) and low nutrition concentrations (total nitrogen < 9 μg/L and total phosphorous < 7 μg/L). Methylmercury in surface water is under the detection limit of 0.0001 ng/L, and so is total mercury.

The mesocosm experiment consisted of four treatments (six replicates for each treatment): 1. Control treatment (“C”, ambient temperature); 2. An elevated temperature treatment (“T”, 3 °C above control temperature average); 3. Browning treatment (“B”, with weekly addition of tDOM solution until one week before the end of the experiment); 4. Mixture of browning and elevated temperature treatment (“TB”, simulated warming and browning with both + 3 °C above control temperature average and addition of terrestrial DOC). The mesocosm system was controlled with respect to temperature, dissolved organic carbon (DOC), light exposure, aeration, and nutrient additions during an experimental period of 42 days. Mounted nylon mesh on top of each mesocosm was set up to protect from invasion of external particles, beetles, or birds, while light exposure was kept to a maximum degree. Each mesocosm was also aerated by an air diffuser to prevent stratification inside the barrel during the experiment. Allocation of treatment for each mesocosm was completely randomized.

Each mesocosm was filled with lake water collected using a submerged water pump connected to a pipe with the inlet located 50 m from lake shore at a water depth of between 2 and 10 m. Then each mesocosm was filled with an equivalent volume (2.5 L) of 100 µm filtered lake water, containing similar distributions of plankton communities. Isotopically enriched MeHg tracers as labile aqueous Me^201^Hg and soil extracted DOC complex were added to the surface water to simulate Hg inputs to the water phase from atmospheric deposition and catchment runoff. Details of tDOM extraction and the mesocosm facility can be retrieved in the supplementary information (SI). Given the extremely low MeHg (below detection limit 0.0001 ng/L) in the water column at the initial stage of the experiment from Lake Lunz, we added an initial MeHg spike to achieve a starting MeHg concentration of 0.05 ng/L in the mesocosm, approximately 10 times higher than the instrumental detection limit, also corresponding to previous MeHg spiked mesocosm experiments^35^. It is also suspected that subalpine lake ecosystems will receive more Hg and possibly MeHg input from either atmospheric^40^ or glacier melt under climate change scenarios^41^, which may result in aqueous MeHg concentrations closer to those currently seen in higher latitude systems. It should be noted that our experimental design has a primary focus on shallow-water lakes, which means we take little consideration in water mixing and upwelling of in-situ produced MeHg as in deeper lakes.

### Maintenance of treatments

From the start (1 July 2018) and throughout the study the temperature was 3 °C higher in the temperature elevated (T and TB treatments) than in the non-temperature regulated enclosures (C and B treatments) (Fig. S1). The DOC concentration was initially 2.21 mg/L in the added water of the control (C) treatments from Lake Lunz and gradually increased to 3.32 mg/L by the end of the experiment. Water colour was measured at least once every week spectrophotometrically (420 nm wavelength) and the browning (B) treatment was maintained by adding weekly the extracted tDOM solution weekly to reach a 2–threefold higher DOC compared to the control. The browning treatments (B and TB) contained DOC concentrations up to 5 mg/L, almost doubled compared to the control treatment. All enclosures were found with algal growth on the wall shortly before the end of the experiment. The enclosure walls were scrubbed at the end of the experiment for collecting biomass and sample analyses.

### Sampling and analysis

Throughout the experiment weekly measurements were taken in both filtered (< 0.2 µm) and unfiltered surface water, of MeHg and total Hg concentrations (Table 2 and Fig. S4). The analysis of aqueous MeHg and total Hg was done using a Tekran Model 2700 (pre-programmed to run EPA Method 1630) and Model 2600 (pre-programmed to run EPA Method 1631), respectively. At the same time, weekly samples of surface water were filtered through 0.7 µm GF/F and analysed for DOC concentrations (GE Healthcare TOC (Total Organic Carbon) Analyzer) and absorbance (UV-1700 Pharmaspec UV–VIS Spectrophotometer) (Fig. 1), total nitrogen (N) using a continuous flow analysis instrument (Alliance instruments) (Fig. S2), chlorophyll a (Chl a) by first extracting samples for Chl a using 90% acetone and then analysing the digested filtrate using a fluorescence spectrophotometer (Hitachi, f-7000 Fluorescence Spectrophotometer) (Fig. S3).

We sampled plankton in different size fractions (seston 0.7–40 µm, microplankton 40–200 µm, zooplankton > 55 µm) at the end of the mesocosm experiment. Besides analysing MeHg in both the water columns and organisms, we also calculated bioconcentration factors (BCF: log10 transformed MeHg concentration ratio between MeHg concentrations in the seston or microplankton (ng/g dry weight, d.w.) and the aqueous MeHg detected in the water column (ng/L) that passed a 0.2 µm filter). The plankton samples were analysed for fatty acid composition, especially fatty acid groups relevant for dietary quality, e.g., omega-3 polyunsaturated fatty acids (n-3 PUFA), and omega-6 polyunsaturated fatty acids (n-6 PUFA). Lipids were first extracted using chloroform:methanol (2:1 v/v) from freeze-dried plankton samples and then methylated to fatty acid methyl esters, which were separated and quantified by gas chromatography^46^. The procedure for lipid analyses of plankton samples is described in the Supplementary Information of this paper. We focused on the seston samples collected on GF/F filters with a size fraction between 0.7 and 40 µm, and microplankton samples collected using 40 µm meshes with a size fraction bigger than 40 µm. The sampled microplankton contained mostly colonial and filamentous Cyanobacteria and small zooplankton (Daphnia and rotifers), which were difficult to separate from the co-occurring algae.

### Statistics

The treatment effects on MeHg and PUFA were tested using a fully replicated factorial ANOVA design. Mean effect sizes (± CI) based on Cohen’d^47,48^ calculated from least square regression model for seston and microplankton are shown in Fig. 2. Differences in the measured values of MeHg and PUFA were tested with one-way ANOVA with Tukey HSD post-hoc test (Table 2). All statistics were performed using JMP Pro 15.

## Supplementary Information

Supplementary Information.

## Data Availability

The data sets generated during and/or analysed for this study are available in supplementary information. Further details and description for the data sets are available from the corresponding author on reasonable request.
