# Supplementary material for: Elevated temperature and browning increase dietary methylmercury, but decrease essential fatty acids at the base of lake food webs
Source: Sci Rep. 2021 Aug 19;11:16859. doi: 10.1038/s41598-021-95742-9 (PMC8376977; doi:10.1038/s41598-021-95742-9)
Supplement: Supplementary file 1 — Supplementary Information. [file 41598_2021_95742_MOESM1_ESM.pdf]

Supplementary Information

Elevated temperature and browning increase dietary methylmercury, but decrease essential fatty acids at the base of lake food webs

Pianpian Wu[1,2*], Martin J. Kainz[3,4], Fernando Valdés[5], Siwen Zheng[6], Katharina Winter[3], Rui Wang[6], Brian Branfireun[7], Celia Y. Chen[2], Kevin Bishop[1]

[1] Department of Aquatic Sciences and Assessment, Swedish University of Agricultural Sciences, Uppsala, Sweden

[2] Department of Biological Sciences, Dartmouth College

[3] WasserCluster Lunz- Biologische Station, Lunz am See, Austria

[4] Department of Biomedical Research, Danube University Krems, Krems, Austria

[5] Uppsala University, Uppsala, Sweden

[6] College of Environmental Science and Engineering, Tongji University, Shanghai, China

[7] Department of Biology, Western University, London, Canada

*Corresponding author: pianpian.wu@slu.se

Summary of experimental setup and sample analysis

24 mesocosms of 400 L were set up in a 30-day outdoor experiment and divided equally into four groups with different planned treatments: Control (C), Temperature (T), Brownification (B) and Mixed (TB). In groups T and TB, a heater (Jbl ProTemp s150) was inserted that was used to increase the temperature with the aim of keeping it three degrees higher than in groups B, and C. It worked by automatically shutting on/off when the temperature got too high or low respectively. Furthermore, groups B and TB were also brownified. The process of brownification involved addition of DOC solutions (GF/F filtered, 0.7 μm) that had been extracted from soil. DOC was added to groups B and TB before and during the experiment

(Table S1, Figure S1) and MeHg + inorganic Hg (iHg) was added to all the mesocosm treatments during the experiment (Table S2). Due to photodegradation of MeHg, it was essential that Hg was added on a continuous basis [1]. The initial Hg spike was made on day 7 of the experiment, which continued for a period of 42 days (Table S2).

To keep track of DOC content, regular measurements of DOC-concentration were made (see Table 1). Absorbance data at 254 nm and 420 nm were also used as indicators for DOC quality and water colour, respectively [2,3]. A way of estimating MeHg-uptake into the food web is to compare the amount of MeHg in an unfiltered water sample from a treatment with a water sample filtered through 0.2 µm filter. Measurements from unfiltered water samples contain both dissolved and particulate MeHg, while filtered has only dissolved MeHg. Chlorophyll a (Chl a), nitrogen (N) concentrations were also measured weekly, and throughout the experiment.

1 Experiment setup and maintenance

1.1 Preparation of mesocosm enclosures

All mesocosm enclosures had lids with a circular opening on the top that was covered with nylon nets to prevent external inputs such as insects, leaves or similar material. A tent was also placed over the entire mesocosm site during the second week of the experiment to protect from rainfall and had minimum shading effects differences across all enclosures. For thermal insulation, the mesocosms were wrapped in plastic sheets (246 cm length x 100 cm width) containing standard insulation material before the experiment began.

Each of the mesocosm barrels was fitted with a sensor for continuous temperature measurement monitoring, as well as an aeration unit and an air diffuser to prevent stratification throughout the experiment.

1.2 Establishing mesocosm ecosystems

Phytoplankton was collected by sampling lake water from Lake Lunz at 4-5 m depth and then sieved the water through 100 µm mesh. Then 2.5 L of collected lake water containing this phytoplankton was distributed to each mesocosm.

Zooplankton was sampled from the pelagic zone of lake Lunz, five vertical tows of 55 µm mesh plankton net from around 30 m depth was added to 2 x 10 L buckets of lake surface water. These were distributed among 24 jars with around 100 zooplankton each and further added to each mesocosm.

1.3 Extraction of dissolved organic matter and addition to mesocosms

Extraction of terrestrial-derived dissolved organic matter (tDOM) was made from soil to mimic brownification in lakes. 18 kg of wet soil was dug up from a peatland natural reserve called Leckermoor (47.785203 N, 14.953324 E). Around 2 kg of wet the soil was taken for dry weight analysis. The rest of the soil was then homogenized using 4 mm mesh hand sieves. This also removed sticks, stones, and other larger components in the soil. The sieved material was placed in a 60 L plastic bucket. Two buckets of 1000 g Chelatin resin (Diaion CR11 sodium form, SUPELCO) was also added to facilitate DOC extraction.

At first, around 30 L of lake water was put in the soil mixture. An Etabo drill connected to a concrete mixer from Kaufman was used to mix the soil in the solution. Afterwards, a SWP 850 submerged pump from Einhell was set to mix the solution for at least 2 hours. The solution was mixed for about 20 minutes every hour. The solution was gradually evaporated and additional lake water was poured in to make up for this. In total, around 65 L of lake

water was added. The mixing went on for six days in which both the concrete mixer and pump were efficiently used. Afterwards, 20 L of soil solution was filtered through a 25 µm sieve. The next day, 3 L of the filtered soil solution was centrifuged on an Avanti J-26 XP Centrifuge (Program JA-14, 10 000 Rpm, 10 min) to get rid of larger particulates.
Of the supernatant, one sample was diluted 1:1000 using lake water filtrate (2.7 µm Whatman GF/D 142 cm diameter, GE Healthcare) to then be sequentially filtered first through 1.2 µm (Whatman GF/C 47 mm diameter, GE Healthcare) and subsequently 0.7 µm (Whatman GF/F; 47 mm diameter, GE Healthcare). The resulting filtrate was then analysed by a TOC analyser (Sievers 900, GE Healthcare).

This procedure was then repeated each week to extract fresh tDOM solution throughout the experiment. Extraction from soil solution, hand sieved down to 30 µm, further filtered through filters of 20 µm, 10 µm, prefilter (pore size between 5 and 10 µm), 5 µm, 2.7 µm, 1.2 µm down to 0.7 µm. If filtering was too slow, the solution was diluted, centrifuged and then further filtered. A small volume of 50 mL tDOM sample was saved for DOC analysis prior to every addition.

Table S1. Addition of terrestrial-derived dissolved organic matter estimated in dissolved organic carbon (DOC) concentrations. Assumed average starting concentration for B and TB treatments was around 0.22 mg/L (derived from starting concentrations of C and T treatments on day one). Estimated DOC concentration added was made based upon measured DOC concentrations in extracted tDOM solution. Total DOC added varied from week to week due to lower DOC extraction efficiency approaching to the end of the experiment.

**Day**	**Volume (L)**	**Estimated DOC concentration added (g/L)**	**Estimated total DOC added [g]**	**Measured DOC concentration treatment B (mg/L) (mean±std)**	**Measured DOC concentration treatment M (mg/L) (mean±std)**
**1**	0.64	1.60	1.28	2.69±0.03	2.54±0.10
**7**	0.50	0.23	0.11	2.94±0.39	3.13±0.45
**10**				3.16±0.18	3.00±0.19
**14**	1.00	0.16	0.16	3.65±0.23	3.52±0.18
**17**				3.52±0.25	3.54±0.28
**21**	1.00	0.35	0.35	3.95±0.12	3.95±0.34
**28**	0.45	0.26	0.12	4.11±0.15	4.04±0.33
**35**	1.00	0.27	0.27	4.73±0.18	4.59±0.25
**42**				4.60±0.23	4.47±0.22

1.4 Hg and MeHg preparation and addition

MeHg was received in the form of monomethylchloride (MeHgCl, chemical form $CH_3Cl^{201}Hg$, an organic form of isotopic enriched ^{201}Hg) at a concentration of 6.2 μg/mL (provided by Chemistry Department, Umeå University, Sweden). Dilution was made accordingly to reach a target MeHg concentration of 0.02-0.05 ng/L in the water of each mesocosm. To better integrate MeHg into the base of the mesocosm food web, MeHg was incubated in green algae (*Scenesdesmus sp.*) leachate that had been sonicated and filtered through GF/C filter (pore size 1.2 μm, Whatman, GE Healthcare). Incubation with DOC, which has been done in some other mesocosm studies, results in MeHg binding to DOC molecules, particularly thiol-groups. This was deemed inappropriate for an oligotrophic lake with pH>8. The incubation with a few drops of green algae leachate was chosen to provide a more appropriate form for phytoplankton assimilation of MeHg [4].

In the first two MeHg additions to the mesocosms (Day 7 and 14), $HgCl_2$ at 10 times higher concentration than MeHg was also incubated with MeHg spikes to simulate MeHg and total Hg proportions in natural environments. We made no further additions of $HgCl_2$ after that point, given high dissolved total Hg concentrations could be detected in the extracted tDOM solution (Fig. S5d). This likely added the extra 2-3 ng/L noted in the water column total Hg of the browning and mixed treatments compared to the control and temperature treatments. The MeHg spikes continued on days 21, 28 and 35. Sampling dissolved MeHg and THg in the water column were made shortly after each of the weekly Hg spikes and water samples were sent to Hg lab by courier for analysis. The fact that the dissolved MeHg concentration in the mesocosms was close to the theoretical concentration that would be achieved by dissolving the spike MeHg in the mesocosm suggests that during the week after each MeHg spike addition, the concentration of dissolved MeHg declined to below the detection limit.

Table S2. MeHg and total Hg spikes for the mesocosm experiment. MeHg concentrations after dilution into the mesocosms were estimated values based on spike solution concentration.

Day	Spiked MeHg in algae-DOM-leachate (mL)	Spiked MeHg and total Hg in algae-DOM-leachate (mL)	Estimated MeHg concentration after dilution into the mesocosm (ng/L)	Estimated Hg concentration after dilution into the mesocosm (ng/L)
**7**		15	0.05	0.5
**14**		5	0.02	0.2
**21**	3		0.03	
**28**	4.25		0.02	
**35**	5		0.02	

1.5 Weekly and final sampling

We sampled surface water (depth 0.1-0.5 m from the surface) from the mesocosms during the experiment period on a weekly basis, and biotic samples were taken at the end of the experiment (Fig. S1). MeHg concentrations were measured within 10 minutes after the spike addition.

The regular weekly samples were analysed for DOC, Chl a, nutrients, and Hg (both MeHg and total Hg). We took surface water samples by first filling two PETG bottles (300 ml, brand new or acid washed) from every mesocosm. One of the bottles was sent for Hg analysis directly, the other was filtered through 0.7 µm (Whatman GF/F; 47 mm diameter) and subsequently through 0.2 µm (Whatman GF/F; 47 mm diameter) prior to Hg analysis.

Both the 0.7 µm and 0.2 µm filters were weighed prior to filtering and further saved in 15 mL falcon tubes for Chl a analysis (0.7 µm) and bacteria fatty acid analysis (0.2 µm). Some of the filtrate through 0.7 µm was saved for DOC analysis (glass vial, 30 mL), optical analysis (glass vial, 30 mL) and nutritional analysis (falcon tube, 15 ml). For both unfiltered and filtered 250 mL PETG bottles, 3.5 ml of 35 % HCl acid was added for preservation of the samples.

All mesocosms were mixed thoroughly prior to the final sampling for plankton.

Microplankton samples were collected using a sampling tube of 3 L that pulled water through 200 µm and 40 µm mesh size filter unit, subsequently. Microplankton that was collected

from filtration of 9 L of mesocosm water was saved for Hg and fatty acid analysis and stored in a 50 ml Falcon tube to be freeze-dried. Another set of microplankton collected from filtering 3 L mesocosm water was stored for taxonomical analysis. 3 L of the 40 µm filtrate was further processed with 0.7 µm GF/F filtration in the lab to obtain seston samples. Zooplankton was collected with a 55 µm filter net trawling from the bottom of mesocosm and then hand-picked to separate from algae. All filtering equipment were rinsed with weak HCl acid and DI water in between sampling.

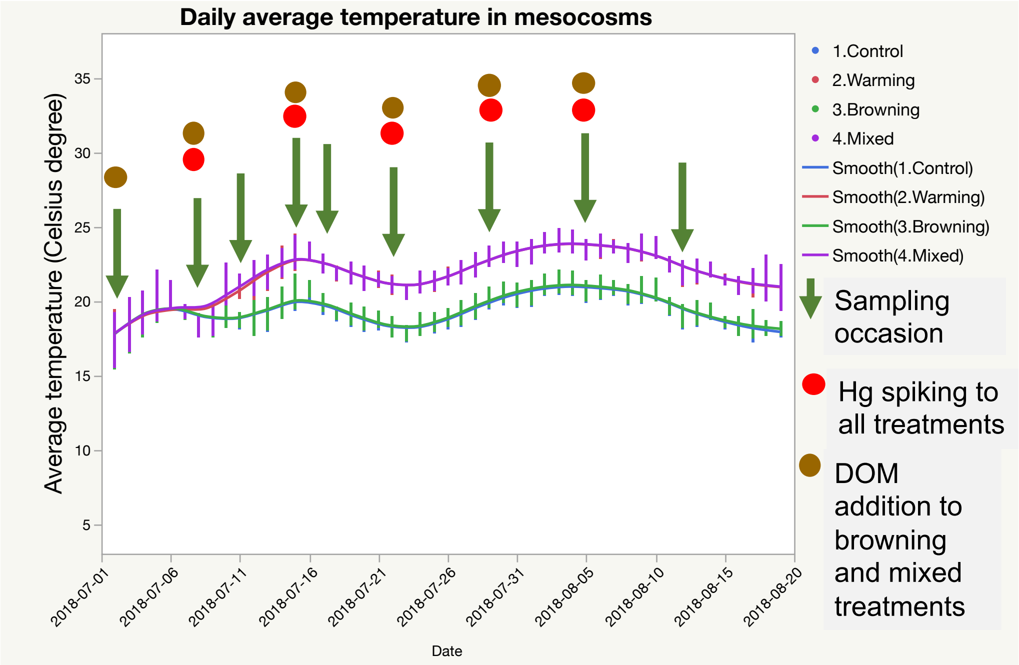

Figure S1. Monitored temperature of the experimental enclosures from a week before (July 2018) to a week after (August 2018) the experiment. Green arrows indicate sampling occasions. Brown dots indicate DOM addition occasions to browning and mixed treatments. Red dots indicate MeHg spiking occasions to all treatments. The additions of DOM and Hg/MeHg were made prior to sampling.

2 Sample analysis

2.1 Analysis of DOC quality and quantity, nutrient, and Chl a

Weekly samples of surface water were analysed for DOC concentrations (GE Healthcare TOC (Total Organic Carbon) Analyzer) and absorbance (UV-1700 Pharmaspec UV-VIS Spectrophotometer). Total nitrogen (N) was analysed using a continuous flow analysis machine (Alliance instruments), results shown in Fig. S2. Chl a was analysed by first extracting Chl a using 90% acetone and then analysing the digested filtrate using a fluorescence spectrophotometer (Hitachi, f-7000 Fluorescence Spectrophotometer). Results of Chl a from weekly samples are shown in Fig. S3.

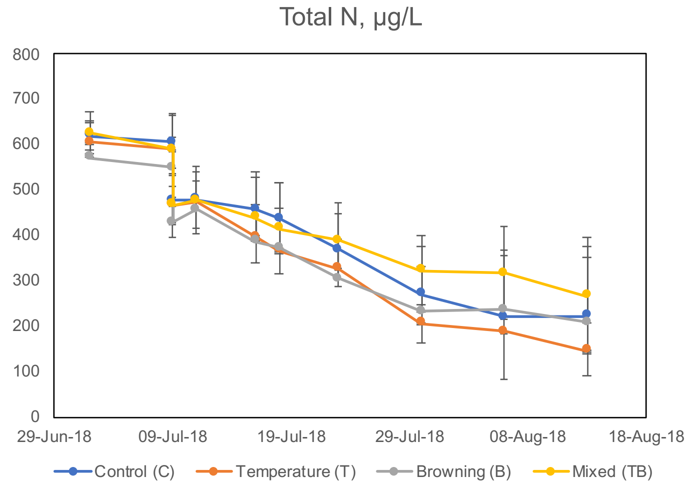

Figure S2. Total nitrogen (N) concentrations over time during the experiment. 09-Jul-18 was the start of spiking MeHg isotopes to the mesocosms.

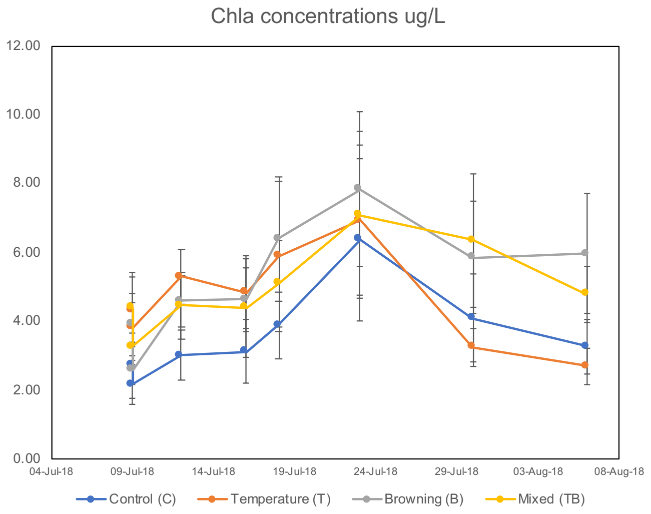

Figure S3. Chlorophyl a (Chl a) concentrations over time during the experiment. Day 7 was the start of spiking MeHg isotopes to the mesocosms.

2.2 Hg and MeHg analysis

Water and biotic samples to be analysed for MeHg and Hg were acidified to 0.4% acid and shipped directly after sampling to the lab of Analytical Services at Biotron Experimental Climate Change Research Centre, University of Western Ontario. The lab is ISO 17025 accredited.

For the water samples, a Tekran Model 2600 was used for measuring total Hg (Method detection limit (MDL): 0.230 ng/L) and a Tekran Model 2700 for MeHg (MDL: 0.006 ng/L). Biotic samples, including freeze-dried GF/F filters and plankton were analysed for total Hg using a Milestone DMA-80 (MDL: 0.04 ng) and MeHg using a Tekran model 2700 (MDL: 0.072 ng/g). The quality control samples for the analysis (initial and ongoing precision and recovery, sample duplicates, sample matrix spikes and %relative percentage differences in sample duplicates and sample matrix duplicate) were all within the test method acceptance criteria. Results of weekly analysis of surface water samples for MeHg and total Hg are shown in Fig. S4 and Fig. S5.

Supplementary Information

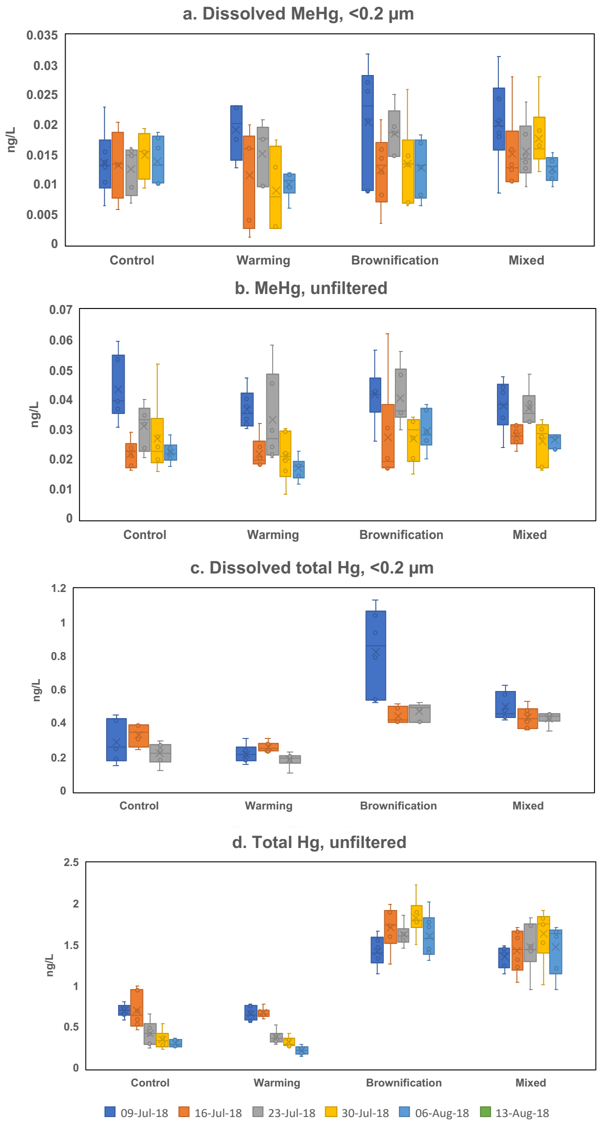

Figure S4. MeHg and total Hg concentrations in surface water samples (a. in dissolved phase <0.2 µm; b. unfiltered water samples) taken directly after spiking MeHg tracers during the experiment. 09-Jul-18 was the start of spiking MeHg isotopes to the mesocosms, 06-Aug-18 was the last MeHg spiking.

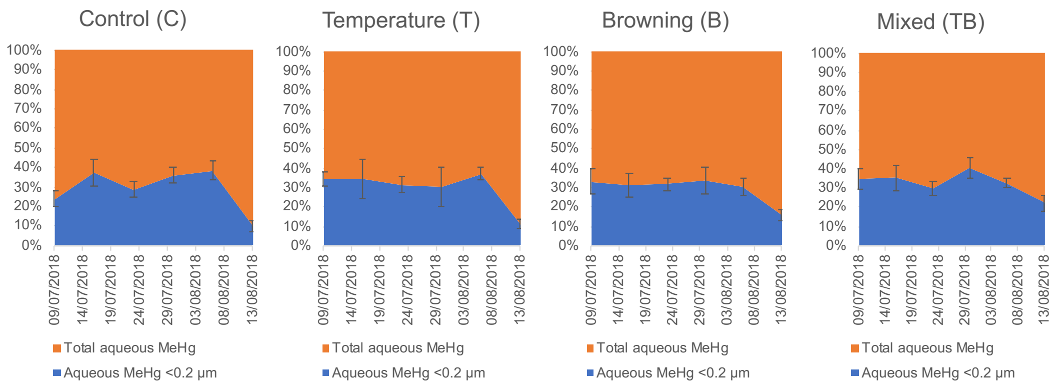

Figure S5. Temporal dynamics of MeHg in surface water samples taken during the experiment shown as aqueous phase <0.2 µm (means±1s.d., in blue) as a proportion of unfiltered water samples (means, in orange).

2.3 Fatty acids analysis

Collected seston samples on GF/F filters and microplankton samples after freeze-drying were analyzed for fatty acids. Fatty acids analyses were performed at WasserCluster Lunz, Austria. Lipids were extracted and analyzed from freeze-dried, homogenized samples of plankton per size-fraction (ca. 1-10 mg dry weight) using chloroform:methanol (2:1 v/v) as described in an earlier publication on fatty acids extraction and analysis [5]. In brief, total lipids were quantified as mass fractions (mg lipids g^{-1} d.w.) gravimetrically using duplicate measurements. Known volumes of total fatty acid extracts were derivatized to fatty acid methyl esters (FAME) using H_2SO_4-methanol (incubated at 50°C for 16 h). FAME were dried under N_2 before being re-dissolved in hexane and run on a gas chromatograph (Thermo Scientific TRACE Gas Chromatograph coupled to flame ionization detection) with a Supelco SP-2560 column used for separation of FAME. FAME were identified by comparison of their retention times with known standards (37-component FAME mix, Supelco 47885-U; bacterial fatty acids, Supelco 47080-U; and the following individual FAME standards: stearidonic acid, O5130 SIGMA™; and n-3 docosapentaenoic acid, Supelco 47563-U) and quantified with reference to seven-point calibration curves derived from 2.5, 50, 100, 250, 500, 1000, 2000 ng μL^{-1} solutions of the FAME standard for each identified FA. FAME were expressed as mass fractions (mg FA g dry wt^{-1}) and as individual FA relative proportions (% of total identified FA).

Supplementary Information

Table S3. Fatty acids analysis results as presented in µg FAME/mg dry weight (dw) per fatty acid compound. Values are stated as average±standard deviation. "n" stands for number of replicates of each treatment. FAME stands for fatty acid methyl esters.

	0.7-40 µm seston				40-200 µm microplankton			
Fatty acids (µg FAME/mg dw)	Control (n=6)	Temperature (n=6)	Brownification (n=6)	Mixed (n=6)	Control (n=6)	Temperature (n=6)	Brownification (n=6)	Mixed (n=6)
14	0.54±0.54	0.98±0.71	0.71±0.17	0.65±0.31	0.47±0.19	0.4±0.29	0.26±0.11	0.26±0.12
iso15:0	0.03±0	0.05±0.04	0.1±0.06	0.08±0.08	0.13±0.05	0.12±0.15	0.07±0.05	0.08±0.04
ai15:0	0.02±0.01	0.04±0.03	0.05±0.02	0.05±0.03	0.07±0.04	0.04±0.02	0.04±0.03	0.04±0.01
14:1n-5		0.01±0.01	0.01±0	0.01±0	0.01±0.01		0.01±0	0.01±0.02
15	0.03±0	0.06±0.04	0.07±0.03	0.05±0.04	0.11±0.04	0.05±0.03	0.04±0.02	0.03±0.01
iso16:0		0.03±0.03	0.05±0.04	0.03±0.05	0.03±0.02	0±0.01		
15:1n-5								
16	2.36±0.42	3.39±1.42	4.76±1.88	3.1±0.45	7.66±2.48	9.68±11.76	3.11±1.7	3.8±1.48
iso17:0		0.04±0.04	0.07±0.04	0.05±0.05	0.01±0.02			0.01±0.02
16:1n-9	0.1±0.03	0.16±0.06	0.27±0.1	0.19±0.07	0.3±0.13	0.28±0.31	0.1±0.04	0.1±0.03
16:1n-7	0.49±0.13	0.66±0.37	0.83±0.36	0.58±0.18	0.89±0.3	0.89±0.69	0.57±0.18	0.57±0.11
17	0.03±0	0.06±0.04	0.09±0.03	0.07±0.06	0.13±0.04	0.11±0.15	0.05±0.03	0.06±0.02
17:1n-7								
18	0.43±0.13	0.61±0.38	0.52±0.14	0.76±0.34	6.6±3.32	8.51±12.03	2.46±1.55	3.16±1.8
18:1n9trans	0.01±0.01		0.02±0.02	0.02±0.03				
18:1n-12	0.12±0.02	0.11±0.1	0.17±0.07					
18:1n-9cis	1.59±0.38	2.33±0.9	2.69±1.19	1.39±0.93	3.27±2.14	4.51±4.76	0.91±0.26	0.85±0.37
18:1n-7	0.26±0.05	0.36±0.16	0.46±0.08	0.38±0.17	0.44±0.16	0.53±0.4	0.38±0.17	0.32±0.06
18:1n-6								
19	0.78±0.4	1.79±1.72	1±0.2	2.55±1.59	11.54±6.41	7.72±5.1	4.28±1.82	2.72±1.73
18:2n-6trans	0.05±0.03	0.02±0.02	0.06±0.03	0.01±0.03	0.21±0.25	0.24±0.38	0.03±0.08	0.04±0.04

	0.7-40 µm seston				40-200 µm microplankton			
Fatty acids (µg FAME/mg dw)	Control (n=6)	Temperature (n=6)	Brownification (n=6)	Mixed (n=6)	Control (n=6)	Temperature (n=6)	Brownification (n=6)	Mixed (n=6)
18:2n-6	1.89±1.05	3.46±1.82	3.68±0.62	2.89±1.13	1.86±0.62	2.16±1.61	0.81±0.21	0.96±0.54
20	0.05±0.02	0.06±0.05	0.06±0.02	0.05±0.01	0.18±0.06	0.17±0.22	0.08±0.04	0.08±0.02
18:3n-6	0.09±0.04	0.14±0.06	0.2±0.11	0.12±0.03	0.32±0.17	0.8±0.6	0.17±0.25	0.09±0.04
20:1n-9	0.01±0.02	0.03±0.05	0.05±0.02			0.04±0.06		0.02±0.02
18:3n-3	2.44±0.97	3.64±1.44	6.1±2.02	3.82±1.26	2.16±0.85	2.55±1.77	1.08±0.43	1.08±0.5
21	0.01±0.01	0.01±0.01	0.01±0	0.01±0.01	0.01±0.01	0.03±0.05	0.02±0	0.01±0.01
18:4n-3	0.23±0.13	0.23±0.08	0.51±0.28	0.26±0.1	0.73±0.37	0.74±0.55	0.23±0.13	0.18±0.15
20:2n-6	0.03±0.01	0.06±0.04	0.11±0.07	0.04±0.03	0.08±0.08	0.09±0.12	0.03±0.05	0.03±0.02
22	0.05±0.02	0.07±0.08	0.09±0.03	0.05±0.01	0.13±0.04	0.11±0.08	0.08±0.04	0.08±0.02
20:3n-6	0.02±0.01	0.04±0.03	0.05±0.03	0.03±0.02	0.02±0.02	0.03±0.03	0.01±0.01	0.01±0.01
22:1n-9		0.08±0.06	0.02±0.03	0.02±0.01	0.11±0.05	0.01±0.02		0.02±0.04
20:3n-3		0.03±0.05	0.19±0.15	0.05±0.04		0.03±0.05		0±0.01
20:4n-6	0.2±0.03	0.35±0.2	0.53±0.24	0.39±0.26	0.17±0.07	0.29±0.26	0.15±0.04	0.15±0.06
23			0.01±0.01					
20:4n-3	0.06±0.05	0.08±0.07	0.13±0.05	0.07±0.03	0.03±0.02	0.03±0.02	0.01±0.01	0.02±0.02
22:2n-6			0.01±0					
24						0.04±0.09		
20:5n-3	0.67±0.17	0.85±0.49	1.6±0.76	0.85±0.45	1.13±0.42	0.98±0.69	0.7±0.28	0.74±0.36
24:1n-9	0.01±0		0.01±0.01			0±0.01	0.01±0.01	
22:3n-3c								
22:4n-6	0.02±0.03	0.03±0.03	0.03±0.03	0.03±0.03	0.16±0.39	0.47±0.93	0.05±0.07	0.06±0.16
22:5n-3	0.03±0.02	0.05±0.04	0.08±0.06	0.04±0.03		0.01±0.02		0±0.01
22:6n-3	0.08±0.03	0.12±0.08	0.13±0.05	0.11±0.07	0.13±0.05	0.1±0.06	0.05±0.03	0.07±0.02

3 Mesocosm condition during the experiment

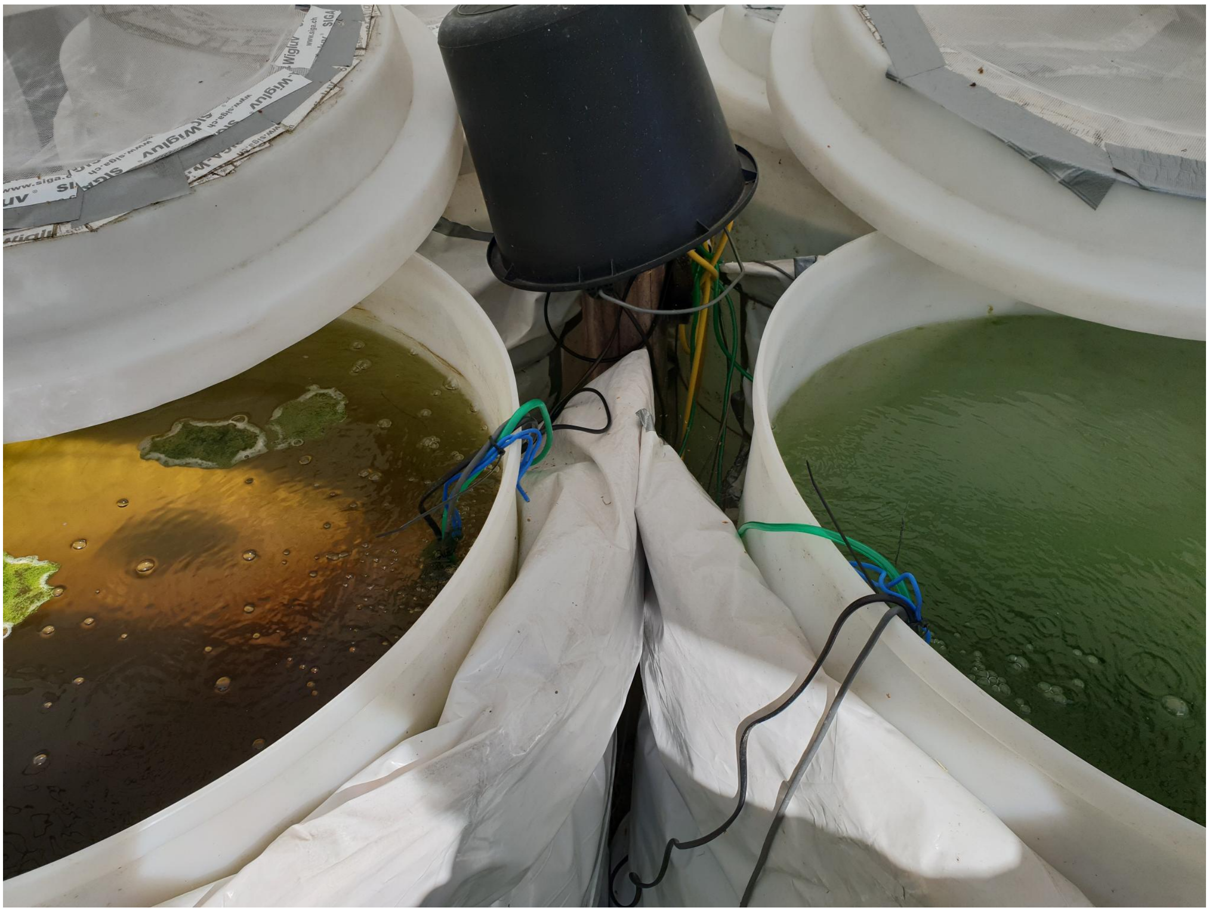

Figure S6. Example of conditions in the experimental mesocosms. Left: Browning (B) treatment mesocosm, floating filamentous algae mat on surface water can be seen. Right: Control (C) mesocosm. Photo taken by K. Bishop about one week before the end of the mesocosm experiment. Photo by Kevin Bishop.

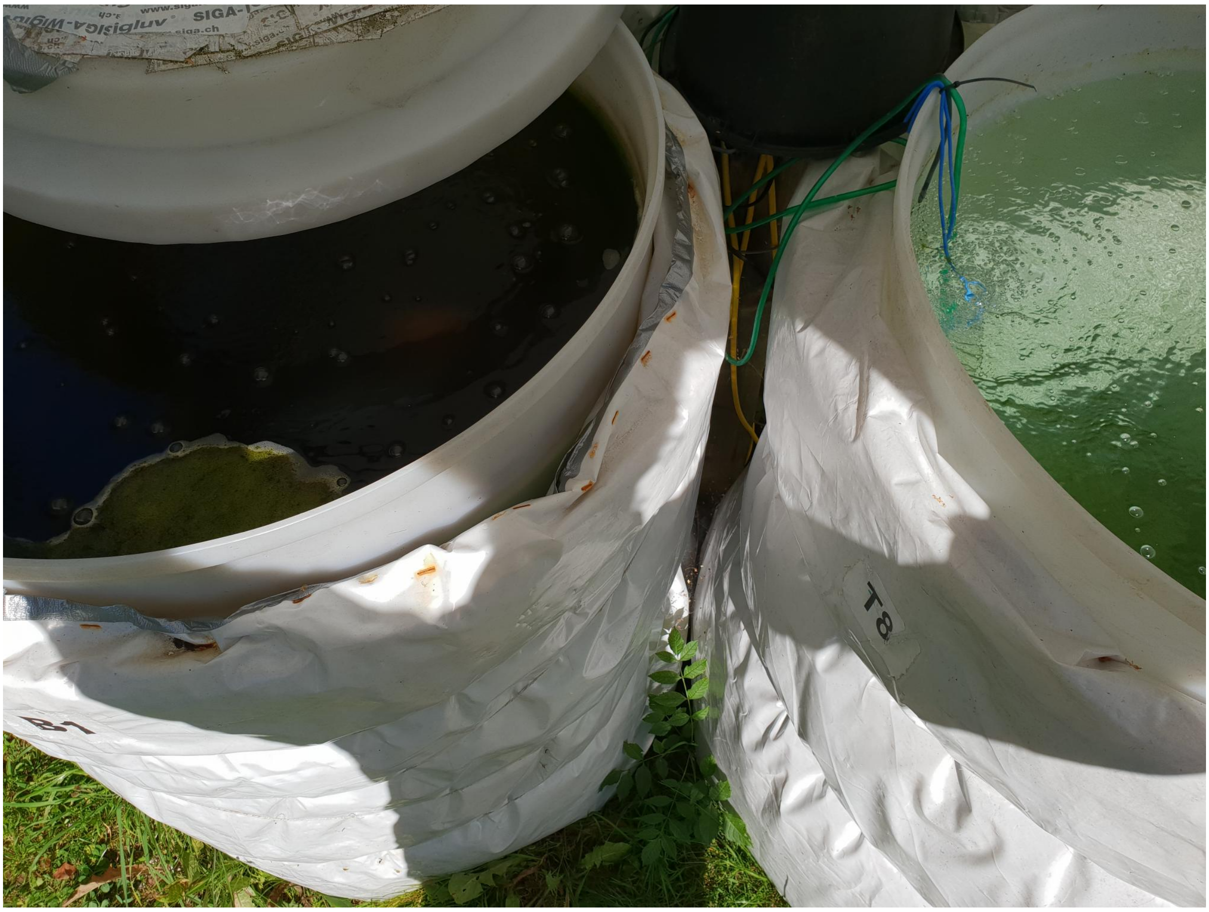

Figure S7. Example of conditions in the experimental mesocosms. Left: Mix (TB) treatment mesocosm, thick floating filamentous algae mat on surface water can be seen. Right: Temperature (T) treatment mesocosm. Photo taken by K. Bishop about one week before the end of the mesocosm experiment. Photo by Kevin Bishop.

References

1 Sellers, P., Kelly, C., Rudd, J. & MacHutchon, A. Photodegradation of methylmercury in lakes. *Nature* **380**, 694-697, doi:10.1038/380694a0 (1996).

2 Brandstetter, A., Sletten, R. S., Mentler, A. & Wenzel, W. W. Estimating dissolved organic carbon in natural waters by UV absorbance (254 nm). *Zeitschrift Fur Pflanzenernahrung Und Bodenkunde* **159**, 605-607, doi:10.1002/jpln.1996.3581590612 (1996).

3 Molot, L. A. in *Encyclopedia of Inland Waters* (ed. Gene E. Likens) 657-663 (Academic Press, 2009).

4 Jonsson, S. *et al.* Differentiated availability of geochemical mercury pools controls methylmercury levels in estuarine sediment and biota. *Nature Communications* **5**, doi:10.1038/ncomms5624 (2014).

5 Heissenberger, M., Watzke, J. & Kainz, M. J. Effect of nutrition on fatty acid profiles of riverine, lacustrine, and aquaculture-raised salmonids of pre-alpine habitats. *Hydrobiologia* **650**, 243-254, doi:10.1007/s10750-010-0266-z (2010).
